# Proficiency in positive vs. negative emotion identification and subjective well-being among long-term married elderly couples

**DOI:** 10.3389/fpsyg.2014.00338

**Published:** 2014-04-28

**Authors:** Raluca Petrican, Morris Moscovitch, Cheryl Grady

**Affiliations:** Department of Psychology and Psychiatry, Rotman Research Institute, University of TorontoToronto, ON, Canada

**Keywords:** emotion recognition, well-being, marriage, Parkinson's Disease, point light walker, older adults

## Abstract

Evidence is accruing that positive emotions play a crucial role in shaping a healthy interpersonal climate. Inspired by this research, the current investigation sought to shed light on the link between proficiency in identifying positive vs. negative emotions and a close partner's well-being. To this end, we conducted two studies with neurologically intact elderly married couples (Study 1) and an age-matched clinical sample, comprising married couples in which one spouse had been diagnosed with Parkinson's Disease (Study 2), which tends to hinder emotional expressivity. To assess proficiency in identifying emotions from whole body postures, we had participants in both studies complete a pointlight walker task, featuring four actors (two male, two female) expressing one positive (i.e., happiness) and three negative (i.e., sadness, anger, fear) basic emotions. Participants also filled out measures of subjective well-being. Among Study 1's neurologically intact spouses, greater expertise in identifying positive (but not negative) emotions was linked to greater partner life satisfaction (but not hedonic balance). Spouses of PD patients exhibited increased proficiency in identifying positive emotions relative to controls, possibly reflective of compensatory mechanisms. Complementarily, relative to controls, spouses of PD patients exhibited reduced proficiency in identifying negative emotions and a tendency to underestimate their intensity. Importantly, all of these effects attenuated with longer years from PD onset. Finally, there was evidence that it was increased partner expertise in identifying negative (rather than positive) emotional states that predicted greater life satisfaction levels among the PD patients and their spouses. Our results thus suggest that positive vs. negative emotions may play distinct roles in close relationship dynamics as a function of neurological status and disability trajectory.

## Introduction

Close relationships constitute a critical ingredient of psychological and physical health (e.g., Beach and O'Leary, 1993; Berscheid and Reis, 1998; Diener and Seligman, 2002). Perhaps unsurprisingly, numerous well-being theories identify fulfilling close relationships as a prerequisite for optimal hedonic balance (e.g., Ryff, 1995; Keyes, 1998; Diener and Biswas-Diener, 2008). Although traditionally relationship researchers focused on the role of negative emotions (e.g., Levenson and Gottman, 1983; Gottman, 1998), there is mounting evidence that positive emotions are crucial in shaping a healthy interpersonal environment (Gable et al., 2006, 2012). Inspired by this research, in the present studies, we sought to examine the link between proficiency in recognizing positive vs. negative emotions and spousal well-being among neurologically intact couples and couples where one partner has been diagnosed with Parkinson's Disease. To situate our present investigation in the literature, we begin by reviewing extant evidence on the role of close relationship quality in health and well-being, followed by a summary of the research on emotions within intimate partnerships, with a focus on social support and care giving circumstances. We conclude the Introduction with an overview of the literature on how emotion recognition proficiency—and thus the ability to provide responsive social support and effective care giving—may be impacted by normal aging and among couples, where one partner is afflicted by neurological conditions, typical of older age, such as Parkinson's Disease.

### Close relationship quality and health

Most of the extant research focuses on the direct pathways through which poor close relationship—most often, marriage—quality can adversely impact well-being and health. For example, relationship turmoil has been found to contribute to psychopathological symptoms such as depression, anxiety, and substance abuse (e.g., Davila et al., 1997; Whisman, 2001; Whisman et al., 2010). Likewise, poorer marital quality and more hostile marital interactions have been linked to abnormal immune responses (Kiecolt-Glaser et al., 1993; Miller et al., 1999), greater cardiovascular reactivity (Morell and Apple, 1990; Ewart et al., 1991; Smith and Brown, 1991; Brown and Smith, 1992; Brown et al., 1998; Newton and Sanford, 2003), higher incidence of cardiac events (Orth-Gomer et al., 2000) and mortality (Eaker et al., 2007). Complementing these findings on the adverse health effects of poor marital quality, there is evidence on the beneficial consequences of greater marital adjustment, such as healthier physiological profiles (i.e., lower blood pressure, Holt-Lunstad et al., 2008), superior immune responses (Kiecolt-Glaser et al., 1987), better cardiovascular health (Baker et al., 2000), and lower mortality following cardiac incidents (among women; Hibbard and Pope, 1993; Rohrbaugh et al., 2006).

### Social support in close relationships

Extending investigations on the direct consequences of marital adjustment on health, there is recent research on the role of marital quality in moderating the effects of non-relationship factors (e.g., work, disease) on physical and psychological well-being. The majority of these studies focused on the stress buffering function of positive marital environments and documented their beneficial effect on rehabilitation among cardiac patients (Wang et al., 2007), recovery from work-related stress (Saxbe et al., 2008) and reduced physiological reactivity to chronic problems at work (Ditzen et al., 2008).

One factor assumed to underlie the stress buffering function of happier marriages is the perception of social support availability, intrinsically linked to being part of a fulfilling intimate partnership (e.g., Kaul and Lakey, 2003). Indeed, there is extensive evidence that such perceptions of emotional support during stressful times constitute a pivotal determinant of physical and psychological health (Cohen and Wills, 1985; Cohen, 1988; Lakey and Cassady, 1990; Uchino et al., 1996; Sarason et al., 1997; Kaul and Lakey, 2003).

Intriguingly, though, perceptions of social support availability are only loosely based on the reality of actual support received during challenging times (e.g., Haber et al., 2007). Instead, they appear to be more heavily rooted in enacted support from others during propitious times (Gable et al., 2012). Moreover, it seems that only the latter is consistently linked to benign personal and relational outcomes (e.g., Gable et al., 2006, 2012), whereas enacted support from others during trying times is only sparsely associated with favorable outcomes, being occasionally even linked to adverse consequences (e.g., Collins et al., 1993; Bolger et al., 2000; Kaul and Lakey, 2003).

The apparent paradox of such findings has been illuminated by studies documenting that it is only provision of social support, perceived to be responsive by the recipient, that contributes to the latter's well-being and feeds his or her perceptions that a close other may be available for assistance during stressful times (Maisel and Gable, 2009; Gable et al., 2012). Provision of responsive support is reportedly easier during auspicious, rather than inauspicious, times, since any visible assistance during the latter periods may only augment the recipient's emotional turmoil by indirectly highlighting his/her inadequacy in coping with the stressor and rendering salient feelings of indebtedness to the support provider (Bolger et al., 2000; Gleason et al., 2003; Shrout et al., 2006). Taken together, the findings reviewed above thus imply that the stress buffering function of close relationships, instantiated in perceptions of emotional support availability during trying times, may be most dependent upon the intimates' ability to read each other's emotions accurately and thus provide each other with responsive support, particularly during favorable times.

### Negative emotions in care giving and receiving

Spousal disease and disability create a dyadic environment in which negative emotions can flourish. Indeed, there is evidence that under such circumstances, it is not only the diseased who struggles with negative emotions, but also the care giver who must manage the challenges of both actively helping his/her ill spouse and dealing with potentially long “on-call” hours (Poulin et al., 2010). Importantly, though, the challenges of attending to an ill spouse are not the sole culprit for the significant hedonic and physical health costs incurred by prolonged care giving (Schulz et al., 2009). Instead, it has been proposed that the affective burden of care giving may be also rooted in the extensive exposure to the suffering of a close other and the intrinsic potential for emotion contagion between the care recipient and giver (for a review, see Monin and Schulz, 2009). Indeed, consistent with a suffering contagion model, several studies provided evidence of significant, positive associations in emotional distress between cancer patients and their caregivers (Given et al., 1993; Northouse et al., 2001). Moreover, longitudinal studies have documented the positive link between spousal suffering and subsequent decay in well-being or even development of depression and physical health problems (e.g., cardiovascular disease) in the care giving partner (Revenson and Majerovitz, 1990; Pakenham, 2001; Schulz et al., 2009).

Although witnessing the suffering of a close other may incur significant hedonic and physical health costs to the care givers, expression of negative emotions by care recipients is nonetheless a key component of effective care giving, because it conveys the need for support and thus enables the caregiver to be more responsive to a care recipient's needs (cf. Monin and Schulz, 2009). Consistent with this interpretation, there is evidence that expression of “vulnerable” negative emotions, such as fear, sadness, anxiety, predicts less caregiver stress (at least among female caregivers, Monin et al., 2009). Indeed, it seems plausible to posit that within a care giving context, the ability to accurately recognize negative emotions may be a critical asset for both the care giver and the care recipient. On one hand, accurate identification of a care recipient's negative emotions may enable the care giver to direct his/her efforts toward maximizing the patient's well-being. Complementarily, accurate identification of a caregiver's negative emotions may aid the care recipient in gauging the impact of his/her illness and adjust behavior (where possible) to reduce care giver burden and, thus, foster a more positive dyadic climate.

### Emotion recognition and aging

An accumulating body of research suggests that, despite the increasing importance placed on emotions in older adulthood (Carstensen, 1992; Mather and Carstensen, 2005), the ability to decode them accurately decays with advancing age (e.g., Ruffman et al., 2009). The bulk of the investigations to date focused on the recognition of facial emotional cues and documented most consistent age-related deficits in the identification of negative emotions, specifically fear, anger, and sadness (Malatesta et al., 1987; Moreno et al., 1993; MacDowell et al., 1994; Brosgole and Weisman, 1995; MacPherson et al., 2002, 2007; Phillips et al., 2002; Calder et al., 2003; Sullivan and Ruffman, 2004a,b; Wong et al., 2005; Keightley et al., 2006; Isaacowitz et al., 2007; Sullivan et al., 2007; Suzuki et al., 2007; Henry et al., 2008; Orgeta and Phillips, 2008; for a recent meta-analysis, see Ruffman et al., 2008).

More recent studies, using a broader array of stimuli, suggest that age-related deficits in emotion identification are not restricted to the decoding of facial cues, but also extend to auditory and postural affective cues (e.g., Brosgole and Weisman, 1995; Montepare et al., 1999; Wong et al., 2005; Ruffman et al., 2009). Moreover, beyond the well-documented global age-related decline in reading emotional expressions, there is also evidence of some modality-specific patterns of impairment. For example, although older adults are reportedly most impaired at recognizing negative emotions from facial cues (cf. Ruffman et al., 2008), they seem to experience greater difficulties with recognizing auditory cues of positive, rather than negative (e.g., fear), emotions (Wong et al., 2005). Similarly, whole-body cues of positive emotions are less legible to older, relative to younger, adults, although age-related deficits in deciphering postural cues have also been detected in response to negative emotions, most notably, sadness, and anger (Montepare et al., 1999; Ruffman et al., 2009).

### Emotion expression and recognition in parkinson's disease

Deficits in emotion recognition are also a hallmark of psychopathological conditions, typical of older age, such as Parkinson's Disease (PD). PD is a degenerative neurological disorder with a prevalence of 1/1000 (Peto et al., 1995). Uncommon before age 40, PD affects around 1% of people over 60 and around 2% of people over 80 (MacPhee and Stewart, 2007). The clinical signs of PD are primarily motor and include slowness of movement, rigidity, resting limb tremors, and postural and balance problems (Ferguson et al., 2008). Recently, though, there has been increasing recognition that despite their salience, motor symptoms are not the only clinical features of PD, which often also is characterized by cognitive deficits (Pillon et al., 1996) and affect dysregulation (Marsh, 2000).

Consistent with the clinical emphasis on motor symptoms, most of the extant empirical investigations on emotion in PD focused on the expressive deficits associated with the disorder. There is thus evidence that PD patients exhibit impairments in producing spontaneous (Buck and Duffy, 1980; Katsikitis and Pilowsky, 1988, 1991; Smith et al., 1996) and, to a somewhat lesser degree, voluntary (Simons et al., 2004) emotional expressions. Such expressive deficits constitute the likely cause of why they tend to be misunderstood and poorly evaluated by their interlocutors (Ellgring et al., 1993), even when the latter are health care professionals (Pentland et al., 1987, 1988). Of note, the motor difficulties associated with PD render the patients least apt at expressing positive emotions. Thus, in the laboratory, PD patients have been found to produce less legible voluntary facial expressions of happiness (Simons et al., 2004). Likewise, outside the laboratory, the facial movement difficulties, which typify PD, cause the patients' spontaneous smiles to be misread as “unfelt” (i.e., inauthentic because of a lack of accompanying cheek raises, Pitcairn et al., 1990).

Complementing the aforementioned investigations on the expressive deficits associated with PD, there is a recently growing body of research on the patients' impairments in decoding emotions. Most of these studies focused on recognition of facial or prosodic cues and provided evidence of marked deficits among PD patients, relative to controls (e.g., Kan et al., 2002; Pell and Leonard, 2003; Yip et al., 2003; Dujardin et al., 2004; Suzuki et al., 2006; Lawrence et al., 2007; Ariatti et al., 2008; Clark et al., 2008; Dara et al., 2008). Indeed, in a recent meta-analysis of the literature on emotion recognition in PD, Gray and Tickle-Degnen (2010) concluded that there is a robust link between PD and impaired recognition of both auditory and facial emotional cues, with deficits being particularly salient for negative emotions.

Despite compelling evidence that PD may incur significant emotion recognition deficits (Gray and Tickle-Degnen, 2010), some inconsistencies do exist in the literature. For example, although robust (adverse) effects of PD on (negative) emotion recognition have been documented among un-medicated, hence, somewhat paradoxically, early stage patients (Sprengelmeyer et al., 2003; Buxton et al., 2013), such impairments have been somewhat harder to detect among medicated patients in the later stages of PD (e.g., Adolphs et al., 1998; Assogna et al., 2010). Moreover, although the bulk of the evidence to date implies that PD may lead to most marked deficits in reading negative emotional cues (Gray and Tickle-Degnen, 2010), a recent study suggests that this may be an artifact of the affective stimuli used by prior research, since PD-related deficits in reading positive emotional cues have been detected when subtler affective expressions are employed (Buxton et al., 2013).

In sum, despite some inconsistencies, extant literature suggests that PD incurs significant deficits both in expressing and decoding affective cues. Interestingly, although deciphering of negative emotions may pose greatest difficulties to PD patients (cf. Gray and Tickle-Degnen, 2010, but see also Buxton et al., 2013), it appears that it is deficient expression of positive emotions that may afflict their social life the most (Pitcairn et al., 1990; Simons et al., 2004).

### Present research

The purpose of the present research was to investigate the link between individual differences in the ability to identify positive vs. negative emotions and a spouse's well-being among neurologically intact elderly couples (Study 1), as well as in an age- and marriage length-matched clinical sample, comprising married couples in which one spouse had been diagnosed with PD (Study 2).

Prior findings suggest that it is only provision of responsive support to a spouse during a positive event that is unambiguously linked to greater recipient well-being (cf. Gable et al., 2012). In contrast, provision of effective support to a spouse during negative events is not only difficult to enact, but even when perceived by the recipient to be highly responsive, it can still not buffer against the deleterious hedonic consequences associated with negative events (although responsive social support is associated with beneficial *relational* outcomes, see Study 2, Gable et al., 2012). Consequently, we hypothesized that among neurologically intact couples, higher levels of spousal well-being would be linked to greater proficiency in identifying positive, rather than negative, emotions. Indeed, we reasoned that those who are “experts” at recognizing positive emotions would be better skilled at reading their spouse's emotional reactions during a positive event and, thus, be in a better position to respond in a manner that would foster the spouse's well-being (cf. Maisel and Gable, 2009; Gable et al., 2012). In contrast, we reasoned that proficiency in identifying negative emotions, and, thus, presumably greater ability to respond effectively to a spouse during negative events, would evidence a weak (if any) relationship to the spouse's well-being.

Complementarily, prior research with couples, in which one partner struggles with a severe disease or disability, suggests that it is the ability to recognize and respond appropriately to negative emotions that may be a critical determinant of dyadic and individual well-being (cf. Monin et al., 2009). Indeed, on one hand, a care giver's ability to read accurately the patient's negative emotions may put him/her in a better position to provide the most responsive and efficient support (cf. Monin and Schulz, 2009). On the other hand, a care recipient's ability to recognize a care giver's negative emotions may allow him/her to adjust behavior (where possible) to minimize care giver burden. Thus, it seemed plausible that in our clinical sample, in which one of the spouses was evidencing increasing levels of disability and, thus, dependence on the care giving partner, the ability to identify accurately negative emotional cues may be as important (if not more important) a contributor to spousal well-being as the ability to identify positive emotions.

For our clinical sample, we opted to focus on PD patients and their spouses for several reasons. First, because it is a neurodegenerative disorder, PD challenges both patients and their spouses to cope with increasing (rather than stable) levels of patient disability, which, arguably, hinders both the patients' and their spouses' ability to habituate to their (ever changing) life circumstances. We thus reasoned that longer disease duration may render expression of negative emotions by the patients increasingly informative for their care givers because with increasing disability, patients become more dependent on their care givers to help them relieve their distress. Consequently, the PD sample allowed us to test the hypothesis that, consistent with the posited adaptive function of negative emotion expression by care recipients (Monin et al., 2009), spousal care givers would demonstrate increased proficiency in decoding negative emotional cues, an advantage that may accentuate with more years from disease onset and greater patient disability.

Second, PD is reportedly associated with deleterious effects on facial affective expressivity (Buck and Duffy, 1980; Katsikitis and Pilowsky, 1988, 1991; Smith et al., 1996), particularly the production of facial positive emotional cues (Pitcairn et al., 1990; Simons et al., 2004). Consequently, inclusion of the PD patients and their spouses allowed us to test whether, in line with our proposed critical role of positive emotion proficiency in fostering spousal well-being, a spouse's declining ability to produce positive emotional cues would be “compensated” by the other's spouse's increasing proficiency in decoding them. Although such effects may generalize across modalities, we reasoned that we may be particularly likely to find evidence of them in modalities that are relatively less affected by PD, such as the postural domain (see below). Finally, inclusion of the PD patients and their spouses in our research also allowed us to extend the literature on emotion perception in PD by examining whether the patients would exhibit deficits in reading postural emotional cues, similar to the ones previously documented for facial and auditory emotional cues (Gray and Tickle-Degnen, 2010).

To assess the cognitive and affective components of well-being, respectively, we had participants in both studies fill out two validated self-report measures (Diener et al., 1985; Gere et al., 2011). To assess individual differences in emotion recognition, we had them complete a point light walker task (Heberlein and Saxe, 2005; Atkinson et al., 2007). We chose this measure for a couple of reasons. First, there is recent evidence that individual differences in performance on this task may be a good indicator of social expertise, since they have been found to be uniquely predictive of individual differences in higher-order sociocogntive processes (e.g., false belief reasoning, Phillips et al., 2011), involved in extracting and updating multiple aspects of social information (Phillips et al., 2011). Second, to the best of our knowledge, despite accumulating evidence on the emotion recognition deficits associated with PD (Gray and Tickle-Degnen, 2010), there is still a dearth of research on the potential PD-associated impairments in decoding postural (rather than facial or auditory) emotional cues. Consequently, we reasoned that probing any potential deficits in reading whole-body emotional cues in PD may be a valuable extension of the literature.

## Study 1

### Methods

#### Participants

Thirty-seven elderly couples, married between 18 and 56 years (*M* = 42.22, *SD* = 9.09, provided informed consent in accordance with the ethical guidelines of the Research Ethics Board at the University of Toronto [women's age: *M* = 69.06 (*SD* = 5.67); men's age: *M* = 72.27 years (*SD* = 6.07)]. All were native English speakers or had lived in Canada and used English as the primary language for at least 30 years. Prior to their laboratory visit, potential participants underwent a phone screening interview, conducted by a senior research assistant, associated with the study. Specifically, they were asked whether (a) they ever had a stroke, tumor, neurological disease, concussion, depression, seizure, head injury, aneurysm, learning disability, psychiatric illness, epilepsy; (b) they had ever been in a serious car accident and/or hit their head badly and/or been unconscious; and (c) what medication (if any) they take on a regular basis. Potential participants who responded “yes” to any of the questions at points “a” and “b” or reported that they were taking psychotropic medication on regular basis were excluded from participating (for the demographic information summary, see Table 1).

**Table 1:** **Demographic information for the Study 1 and 2 samples**.

	**Study 1**	**Study 2**
1. Marriage length	42.22 ± 9.09	39.28 ± 11.67 years
2. Females	Age = 69.06 ± 5.67 years	Age = 67.77 ± 9.95 years
3. Males	Age = 72.27 ± 6.07 years	Age = 68.39 ± 10.22 years
4. PD patients		12 males; Age = 69.75 ± 9.34 years 6 females; Age = 66.00 ± 10.99 years
5. PD spouses		12 females; Age = 68.72 ± 9.76 years 6 males; Age = 65.67 ± 12.24 years
6. Years from PD symptom onset		8.06 ± 3.89 years

*The above information is based on data from 37 couples (Study 1) and 18 couples (Study 2)*.

#### Measures

***Pointlight walker task.*** To assess participants' ability to read emotions from whole body postures, we presented them with a set of 38 movies, ranging in length from 4 to 14 s, obtained upon request from Andrea Heberlein (see also Heberlein and Saxe, 2005; Atkinson et al., 2007). According to Andrea Heberlein, this set of stimuli most consistently elicited emotionally valenced judgments from neurologically intact participants (i.e., the actor in the clip was perceived to express an emotion, rather than being neutral). The stimuli featured professional or student actors, 3 males and 3 females, who walked across the frame of a movie camera from left to right, with movement patterns intended to convey specific emotions (i.e., anger, fear, happiness, or sadness). Small lights were attached to the actors' wrists, ankles, knees, elbows, outer hip, waist, outer shoulder, and head; they were filmed in the dark, so that only the moving lights were visible. At the end of each movie clip, participants were requested to rate on a 7-point scale (from 1 not at all to 7 extremely) the extent to which the actor expressed each of the four emotions (i.e., fear, sadness, anger, happiness) or s/he was neutral (i.e., expressed no emotion at all). Taking our cue from Adolphs et al. (1998) and Dujardin et al. (2004), we chose to use the emotion rating profile, rather than categorical labeling of each movie within one emotion, to account for the fact that subtler, realistic cues are unlikely to be emotion-pure (i.e., other emotions are likely to be expressed together with the targeted one).

***Data scoring.*** For each clip, across all participants, we averaged separately the scores provided for each of the four emotions tested to create the sample's “agreed upon” ratings of each movie. Subsequently, for each participant and for each of the four scrutinized emotions, we created a “deviance” score as the absolute value of the difference between the sample's average rating of the movie on the given emotion and the respective participant's rating. Then, for each participant, we averaged these “deviance” scores across all movies, within each of the four tested emotions. For ease of interpretation, we then subtracted each “deviance” score from 1, so that for the resulting scores, greater values would reflect better recognition performance. Consequently, indicative of our ultimate interest in a participant's ability to accurately read his/her spouse's emotions, we have operationalized “accuracy” in emotion recognition as (greater) agreement between a participant's emotion ratings and the sample's mean emotion ratings. Our rationale was that, beyond any idiosyncratic effects, a significant predictor of whether a participant would successfully decode his/her spouse's emotions is his/her adherence to the affective vocabulary of those most demographically similar to his/her spouse. We also kept the raw values of the difference between a participant's rating of a movie on a given emotion and the sample's corresponding rating. These scores, averaged across all movies, indicated the direction of a participant's leaning on a given emotion judgment (i.e., over- or under-estimation of the intensity of an emotion, relative to the sample).

***Subjective well-being.*** Participants completed a 7-point Likert-type life satisfaction measure (e.g., “I am satisfied with my life.”; Diener et al., 1985; α = 0.86) and a positive and negative affect scale (e.g., “In general, I felt bad.”; (Gere et al., 2011); α = 0.87 and 0.90, respectively). To compute an index of hedonic balance, we subtracted participants' average score on the negative affect scale from their average score on the positive affect scale. Consistent with Diener's (1984) proposal that life satisfaction and hedonic balance constitute aspects—cognitive and affective, respectively—, of the same construct, i.e., subjective well-being, their indices were moderately correlated, r_(72)_ = 0.45, *p* < 0.01.

#### Procedure

The study period was comprised of two 90-min sessions separated by a 30-min lunch break. Upon arriving at the lab, spouses were taken to separate testing rooms, where they remained for the duration of the two study sessions, reuniting during the break. At the beginning of the first session, partners were asked to fill out a larger questionnaire package that included the life satisfaction and affective well-being scales. During the second session, they completed the point light walker task. All measures and tasks were administered in this fixed order across all participants.

### Results

#### Data preparation

Due to the dependency in our couples' data, hierarchical linear regression models were regarded as the most appropriate statistical approach (HLM 7.01, Raudenbush et al., 2013). HLM produces essentially the same parameter estimates as simple linear regression, but uses more appropriate estimates of standard errors to test statistical significance. Following the recommendations of Campbell and Kashy (2002) for analysis of dyadic data in HLM, we investigated the interrelationships among our various measures by running fixed slopes regression models. As in simple regression, the outcome variable was uncentered. All predictors were mean-centered. In all the reported analyses, we included both actor and partner variables for all the predictors (all the reported results are unchanged if only the partner variables are introduced as predictors). Because the data violated normality assumptions, we report the robust standard error estimates for all the analyses conducted (cf. Hox, 2002).

#### Preliminary analyses

***Gender effects.*** A One-Way ANOVA with gender as a fixed factor revealed that males exhibited lower accuracy in reading negative emotions (*M* = 0.24, *SD* = 0.67) relative to females (*M* = 0.50, *SD* = 0.30), *F*_(1, 73)_ = 4.49, *p* < 0.05, which was apparently due to their tendency to overperceive the amount of negative emotions portrayed in the clips (*M* = 0.18, *SD* = 0.67) relative to females (*M* = −0.17, *SD* = 0.50), *F*_(1, 73)_ = 6.47, *p* < 0.05. At the level of discrete emotions, this gender effect appeared to be driven by the males' tendency to overperceive sadness (*M* = 0.35, *SD* = 1.07), *F*_(1, 73)_ = 10.75, *p* < 0.01, and anger (*M* = 0.38, *SD* = 1.14), *F*_(1, 73)_ = 8.07, *p* < 0.01, relative to females (*M* = −0.31, *SD* = 0.60 for sadness and *M* = −0.21, *SD* = 0.52 for anger). In light of these gender differences, all the reported analyses controlled for gender, coded as −1 for males and 1 for females.

***Actor-partner correlations.*** Using a two-level HLM model with individuals (level-1) embedded in couples (level-2) and gender as a control variable, we found a significant positive association between the spouses' satisfaction with life, *b* = 0.49, *SE* = 0.11, *t*_(35)_ = 4.45, *p* < 0.01, but not hedonic balance levels, *b* = 0.15, *SE* = 0.15, *t*_(35)_ = 1.05, *p* = 0.30. Likewise, there was evidence that spouses tended to be similar not only with respect to their global ability to accurately identify the emotions portrayed in the movie clips, *b* = 0.45, *SE* = 0.23, *t*_(35)_ = 1.96, *p* = 0.06, but also with respect to their ability to accurately identify discrete emotions, specifically, happiness, *b* = 0.42, *SE* = 0.14, *t*_(35)_ = 2.93, *p* < 0.01, and fear, *b* = 0.30, *SE* = 0.12, *t*_(35)_ = 2.47, *p* = 0.02 (for the intercorrelations among the Study 1 measures, see Table 3).

#### Hypothesis testing

***Positive emotions and partner hedonic balance.*** Taking our cue from previous findings that provision of responsive support to a close partner during a positive event is a core contributor to the recipient's well-being (e.g., Gable et al., 2012), we tested whether accuracy in identifying positive emotions—and, thus, plausibly, superior ability to respond appropriately to circumstances giving rise to such affect—would predict a spouse's self-ratings of hedonic balance (i.e., the affective component of well-being, cf. Diener, 1984). Results of a regression analysis, predicting an actor's hedonic balance from his/her partner's ability to identify happiness, as well as the actor's own proficiency in detecting happiness disconfirmed our hypothesis, since neither predictor was found to exert a statistically significant effect, *b* = 0.43, *SE* = 0.28, *t*_(34)_ = 1.57, *p* > 0.12 (the partner's ability to identify happiness) and *b* = 0.06, *SE* = 0.19, *t*_(34)_ = 0.30, *p* = 0.77 (the actor's ability to identify happiness) (for the full model, see Table 2).

**Table 2:** **Parameter estimates for the HLM analyses predicting an actor's hedonic balance and life satisfaction from his/her partner's proficiency in identifying positive emotions in Study 1**.

**Fixed effect**	**Coefficient**	***SE***	***t*-value (dfs)**
**OUTCOME: ACTOR_HEDONIC_BALANCE**			
For overall INTERCEPT, β_0_	2.71	0.17	16.00 (36)^**^
For ACTOR_GENDER slope, β_1_	0.06	0.15	0.42 (34)
For PARTNER_HAPPINESS_RECOGNITION slope, β_2_	0.43	0.27	1.57 (34)
For ACTOR_HAPPINESS_RECOGNITION slope, β_3_	0.06	0.19	0.30 (34)
**OUTCOME: ACTOR_SATISFACTION_WITH_LIFE**			
For overall INTERCEPT, β_0_	5.51	0.11	49.47 (36)^**^
For ACTOR_GENDER slope, β_1_	−0.04	0.07	−0.55 (34)
For PARTNER_HAPPINESS_RECOGNITION slope, β_2_	0.40	0.12	3.31 (34)^**^
For ACTOR_HAPPINESS_RECOGNITION slope, β_3_	0.23	0.19	1.20 (34)
**OUTCOME: ACTOR_SATISFACTION_WITH_LIFE**			
For overall INTERCEPT, β_0_	5.51	0.10	56.61 (36)^**^
For ACTOR_GENDER slope, β_1_	−0.05	0.07	−0.75 (33)
For PARTNER_HAPPINESS_RECOGNITION slope, β_2_	0.31	0.12	2.50 (33)^*^
For ACTOR_HAPPINESS_RECOGNITION slope, β_3_	0.21	0.19	1.15 (33)
For ACTOR_HEDONIC_BALANCE slope, β_4_	0.21	0.06	3.77 (33)^**^

* p < 0.05;

** *p < 0.01*.

**Table 3:** **Intercorrelations among the Study 1 measures**.

	**1**	**2**	**3**	**4**	**5**	**6**	**7**	**8**	**9**	**10**	**11**	**12**
1. Spouse 1 happiness recognition	−											
2. Spouse 1 fear recognition	0.33^**^	−										
3. Spouse 1 sadness recognition	0.24^*^	0.55^**^	−									
4. Spouse 1 anger recognition	0.15	0.36^**^	0.33^*^	−								
5. Spouse 2 happiness recognition	0.42^**^	0.32^*^	0.17	−0.03	−							
6. Spouse 2 fear recognition	0.32^*^	0.30^*^	0.15	0.07	0.33^**^	−						
7. Spouse 2 sadness recognition	0.17	0.15	0.07	0.10	0.24^*^	0.55^**^	−					
8. Spouse 2 anger recognition	−0.03	0.07	0.10	0.05	0.15	0.36^**^	0.33^*^	−				
9. Spouse 1 SWLS	0.15	0.21^**^	0.12	0.07	0.26^**^	0.19	−0.04	−0.06	−			
10. Spouse 1 AWB	0.08	0.22	0.13	0.01	0.18	0.09	−0.02	−0.05	0.36^**^	−		
11. Spouse 2 SWLS	0.26^**^	0.19	−0.04	−0.06	0.15	0.21^**^	0.12	0.07	0.49^**^	0.12	−	
12. Spouse 2 AWB	0.18	0.09	−0.02	−0.05	0.08	0.22	0.13	0.01	0.12	0.15	0.36^**^	−

* p < 0.05;

** *p < 0.01*.

*N = 74 individuals embedded in 37 couples. To obtain standardized coefficients, correlations were computed using standardized variables in a two-level HLM model, which collapsed across all participants (Level-1), but controlled for gender and accounted for the interdependence in the data provided by the two spouses (Level-2: couple level). The coefficients and significance estimates in the table are based on the robust standard error estimates, provided by HLM. SWLS, satisfaction with life. AWB, hedonic balance*.

***Positive emotions and partner life satisfaction.*** In contrast, results of the analyses involving the cognitive component of well-being (i.e., life satisfaction, Diener, 1984) supported our hypothesis regarding the unique beneficial effect of a partner's proficiency in identifying positive emotions on an actor's well-being. Specifically, results of a regression analysis, predicting an actor's life satisfaction from his/her partner's ability to identify happiness, as well as the actor's own proficiency in detecting happiness revealed a significant effect of the former predictor, *b* = 0.40, *SE* = 0.12, *t*_(34)_ = 3.31, *p* < 0.01, but not the latter, *b* = 0.23, *SE* = 0.19, *t*_(34)_ = 1.20, *p* = 0.24 (for the full model, see Table 2). Indeed, further testifying to the specificity of this effect, a subsequent analysis revealed that a partner's proficiency in identifying happiness remained a significant predictor of an actor's satisfaction with life levels, even after accounting for the actor's hedonic balance, *b* = 0.31, *SE* = 0.12, *t*_(3)_ = 2.50, *p* = 0.02 (for the full model, see Table 2).

#### Post-hoc analyses

***Negative emotions and partner well-being.*** Since prior findings suggest that the partner's responsiveness to an actor's negative affective states may have some impact on the latter's well-being (Study 2, Maisel and Gable, 2009; Gable et al., 2012), we explored whether the partner's accuracy in reading negative emotions would predict an actor's subjective well-being levels. Results of this set of analyses provided no support for this conjecture with regards to either the actor's satisfaction with life, *b* = 0.13, *SE* = 0.24, *t*_(34)_ = 0.55, *p* = 0.59, or his/her hedonic balance, *b* = −0.02, *SE* = 0.28, *t*_(34)_ = −0.07, *p* = 0.95. Likewise, there was no evidence that an actor's greater accuracy in reading negative emotions would be a significant predictor of either his/her satisfaction with life, *b* = 0.31, *SE* = 0.16, *t*_(34)_ = 1.91, *p* = 0.06, or hedonic balance, *b* = 0.39, *SE* = 0.38, *t*_(34)_ = 1.03, *p* = 0.31. Because prior research suggested that expertise in recognizing distinct negative emotions may differentially impact social interactions (e.g., Niedenthal and Brauer, 2012), we investigated whether expertise in decoding fear vs. anger vs. sadness would be distinctly associated with partner well-being. Results of these analyses revealed only a significant link between an actor's proficiency in reading fear and his/her life satisfaction levels, *b* = 0.31, *SE* = 0.12, *t*_(34)_ = 2.66, *p* = 0.01 (all other *p*s > 0.11) (for standardized coefficients, see Table 4).

**Table 4:** **Mean values of happiness and negative emotion recognition accuracy, as well as negative emotion leaning, among spouses of PD patients vs. controls as a function of years from PD symptom onset**.

	**Early PD spouses symptom onset < 5 years**	**Late PD spouses symptom onset > 11 years**	**Controls**
1. Happiness recognition accuracy	0.60	0.26	0.36
2. Negative emotion recognition accuracy	0.32	0.78	0.37
3. Negative emotion leaning	−0.73	−0.19	0.00

*For Happiness and Negative Emotion Recognition Accuracy, respectively, higher coefficients indicate greater accuracy. For Negative Emotion Leaning, lower (i.e., more negative) values indicate greater under-estimation of the amount of negative emotion. The early and late PD groups comprise 4 couples each (8 in total), in which the number of years from PD symptom onset is 1 SD below and above, respectively, the PD sample's average number of years from PD onset*.

***Gender differences in the reported effects.*** Finally, we verified that there were no statistically significant gender differences in the relationship between an actor's proficiency in identifying positive vs. (global/discrete) negative emotions and his/her partner's either satisfaction with life (all *p*s > 0.18) or hedonic balance (all *p*s > 0.05).

In sum, Study 1 provided partial support to our hypothesis that greater expertise in identifying positive emotions would be linked to greater spousal well-being, conceivably because such expertise may render one better skilled at reading his/her spouse's emotional reactions during a positive event and, thus, be in a better position to respond in a manner that would foster the spouse's well-being (cf. Gable et al., 2012). Specifically, we found that a partner's proficiency in identifying positive emotions exhibited a significant association with an actor's life satisfaction, but not hedonic balance. Interestingly, this effect points to a potential interpersonal mechanism underlying the observed spousal similarity in life satisfaction (for similar findings, see Bookwala and Schulz, 1996; Schimmack and Lucas, 2010), since spouses were alike not only with respect to their cognitive well-being levels, but also with respect to their ability to identify positive emotions. Finally, Study 1 also revealed a potential intra-individual mechanism supporting spousal similarity on life satisfaction, since it provided evidence of spousal resemblance on fear recognition ability, which was, in turn, a significant intrapersonal predictor of life satisfaction. We will elaborate on the implications of these findings in the General Discussion.

## Study 2

The purpose of Study 2 was to investigate whether under circumstances that render negative emotions particularly salient and informative, such as spousal disability (Monin and Schulz, 2009; Monin et al., 2009), expertise in decoding negative (rather than positive) emotions would be a stronger predictor of spousal well-being. Moreover, we sought to replicate the Study 1 findings that it is an actor's cognitive, rather than affective, well-being levels that are most susceptible to the influence of spousal proficiency in (here, negative) emotion processing.

In a secondary vein, we also examined whether beyond any adverse effects of PD on the patients' ability to decode postural emotional cues, we would also detect any differences in emotion recognition performance between the patients' spouses and the controls, presumably reflective of the former's adaptations to living with PD. Specifically, we investigated whether in order to compensate for the PD patients' presumed deficits in producing facial expressions of positive emotions (cf. Pitcairn et al., 1990), their spouses would be particularly attuned and, thus, demonstrate increased accuracy in decoding expressions of happiness. Although it is plausible that such an adaptation effect (if it exists) may generalize across modalities, we reasoned that we may be particularly likely to detect it in a modality that is presumably less affected by PD (i.e., by using postural, rather than facial, cues of happiness). Finally, based on the assumption that proficiency in recognizing negative emotions may be a critical asset for care givers (i.e., to minimize care giver stress, cf. Monin et al., 2009), we examined whether relative to controls, PD spouses would also show increased expertise in reading negative emotions, specifically those indicative of vulnerability, such as fear and sadness. In all the reported analyses, the Study 1 participants served as the control group [*t*-test analyses confirmed that there were no significant differences in either age (*p* > 0.10) or marriage length (*p* > 0.28) between the Study 1 and 2 samples].

### Methods

#### Participants and procedure

Eighteen PD patients and their spouses completed the same tasks and measures as the Study 1 participants. The patients were recruited through local newspaper advertisements or through their neurologist, who was affiliated with a teaching hospital associated with the University of Toronto. According to the patients' medical records (released with their consent by their neurologist following the patients' laboratory visit), they were all non-demented and not clinically depressed at the time of testing. Prior to their laboratory visit, the patients' spouses underwent the same phone screening interview as the Study 1 participants, in which they verified that they themselves had no known neurological or cognitive impairments. The patients [6 women, 12 men; mean age = 68.50 years (*SD* = 11.67)] averaged 2.56 (range 1.0–3.0) on the modified Hoehn and Yahr (1967) disability scale. On average, they had received the PD diagnosis 8.06 years (*SD* = 3.89 years; range 2–15 years) prior. Excepting one who developed intolerance to L-dopa six months prior, all were taking dopamine precursor treatments (i.e., L-dopa) to alleviate Parkinsonian symptoms. All patients were tested while on normal dosing schedules. They were tested late morning, approximately 3 h after taking their medication. The spouses' average age was 67.28 years (*SD* = 10.23). The couples had been married between 18 and 53 years (*M* = 39.28, *SD* = 11.67). All were native English speakers or had lived in Canada and used English as their primary language for at least 30 years (for the demographic information summary, see Table 1). Informed consent was obtained from both PD patients and their spouses in accordance with the ethical guidelines of the Research Ethics Boards at the University of Toronto and the University Health Network.

### Results

Preliminary analyses confirmed that all the reported results were unchanged if the patient who became intolerant to L-dopa, and who was thus medication-free during the study period, was kept or eliminated from the sample. Consequently, we opted to keep this patient's data in the analyses. Two PD patients failed to fill out the positive/negative affect scale, whereas one PD spouse failed to complete the satisfaction with life scale. Consequently, all the reported well-being findings are based on data from 18 PD patients and 17 PD spouses (satisfaction with life), as well as 16 PD patients and 18 PD spouses (hedonic balance).

#### Preliminary analyses

Preliminary correlational analyses revealed no statistically significant association between the patients' and their spouses' satisfaction with life, *r*_(16)_ = 0.39, *p* = 0.12, hedonic balance, *r*_(15)_ = −0.32, *p* = 0.24, proficiency in recognizing positive, *r*_(16)_ = 0.15, *p* = 0.56, or negative, *r*_(16)_ = −0.25, *p* = 0.33, emotions.

#### Hypothesis testing

To test our hypotheses, we used the same two-level HLM model as in Study 1 and ran two sets of analyses, comparing the healthy controls to the PD patients and the patients' spouses, respectively. As in Study 1, we controlled for gender (coded as −1 for males and 1 for females) in all the reported analyses. For the comparisons involving the PD patients and the controls, two predictor variables were used: patient status, coded 0 for controls and 1 for PD patients and years from PD symptom onset, which had a value of 0 for controls and a number corresponding to the years from the symptom onset for patients. Likewise, for the comparisons involving the spouses of PD patients and controls, two predictors were employed: patient spouse status, coded 0 for controls and 1 for the spouses of the PD patients and years from PD symptom onset, which had a value of 0 for controls and a number corresponding to the years from the symptom onset for the patients' spouses. As in Study 1, we report the robust standard error estimates for all the analyses conducted.

***Well-being.*** As expected, PD patients exhibited significantly lower satisfaction with life levels compared to their age-matched controls, *b* = −2.40, *SE* = 0.48, *t*_(34)_ = −4.97, *p* < 0.001, although the discrepancy between the two groups diminished with more years from PD symptom onset, *b* = 0.14, *SE* = 0.06, *t*_(34)_ = 2.46, *p* = 0.02. Hedonic balance was also poorer among PD patients, relative to their age-matched controls, *b* = −2.11, *SE* = 0.93, *t*_(34)_ = −2.26, *p* = 0.03, and this effect was impervious to the number of years from PD symptom onset, *b* = 0.16, *SE* = 0.14, *t*_(34)_ = 1.13, *p* = 0.27. In contrast, although spouses of PD patients tended to experience lower satisfaction with life as well, this effect failed to reach conventional levels of statistical significance, *b* = −0.97, *SE* = 0.50, *t*_(34)_ = −1.96, *p* = 0.06. Of note, there was no evidence that spouses of PD patients would exhibit poorer affective balance relative to their age-matched controls, *b* = −1.08, *SE* = 0.93, *t*_(34)_ = −1.16, *p* = 0.25, or that years from PD symptom onset would lead to any significant differences between the two groups in either satisfaction with life, *b* = 0.06, *SE* = 0.07, *t*_(34)_ = 0.87, *p* = 0.39, or hedonic balance, *b* = 0.07, *SE* = 0.12, *t*_(34)_ = 0.60, *p* = 0.55.

***Emotion recognition.***

*PD patients vs. controls*.

*Positive emotions*: We found no evidence that either patient status, *b* = −0.09, *SE* = 0.21, *t*_(34)_ = −0.44, *p* = 0.66, or years from PD symptom onset, *b* = 0.00, *SE* = 0.02, *t*_(34)_ = 0.14, *p* = 0.89, would impact significantly accuracy in identifying positive emotions.
*Negative emotions*: Likewise, there was no evidence that either patient status, *b* = 0.16, *SE* = 0.18, *t*_(34)_ = 0.90, *p* = 0.37, or years from PD symptom onset, *b* = −0.02, *SE* = 0.01, *t*_(34)_ = 1.05, *p* = 0.30, would affect significantly accuracy in identifying negative emotions. Subsequent analyses focused on accuracy in identifying discrete negative emotions yielded similar, non-significant results (all *p*s > 0.33).

*Spouses of PD patients vs. controls*.

*Positive emotions*: Results of this analysis revealed two significant main effects: Spouses of PD patients were more accurate than controls in identifying happiness, *b* = 0.58, *SE* = 0.17, *t*_(34)_ = 3.46, *p* < 0.001, but their advantage diminished with more years from PD symptom onset, *b* = −0.05, *SE* = 0.02, *t*_(34)_ = 2.60, *p* = 0.01 (see Table 4).
*Negative emotions*: Interestingly, we found evidence that spouses of PD patients tended to be somewhat less accurate than controls in identifying negative emotions, *b* = −0.51, *SE* = 0.25, *t*_(34)_ = 2.05, *p* = 0.05, although their “deficit” was reversed with more years from PD symptom onset, *b* = 0.06, *SE* = 0.02, *t*_(34)_ = 2.45, *p* = 0.02 (see Table 4). Follow-up analyses revealed that these effects were due to the PD spouses' tendency to underestimate the intensity of negative emotions (relative to controls), *b* = −0.64, *SE* = 0.28, *t*_(34)_ = −2.31, *p* = 0.03, a tendency that became weaker with longer years from PD symptom onset, *b* = 0.07, *SE* = 0.03, *t*_(34)_ = 2.34, *p* = 0.03 (see Table 4). Nevertheless, there was no evidence that the relatively poorer identification of negative emotions was specific to any of the discrete negative emotions under scrutiny (all *p*s > 0.09).

***Emotion recognition and partner well-being.*** Finally, we tested our hypothesis that among PD patients and their spouses, it would be expertise in decoding negative, rather than positive, emotions that would be a stronger predictor of partner well-being. To this end, we specified a two-level HLM model, with years from PD symptom onset as the level-2 (i.e., couple-level) variable, gender (coded as −1 for males and 1 for females), patient status (coded as −1 for PD patients' spouses and 1 for PD patients), and accuracy in identifying positive vs. negative emotions as the level-1 variables. Both level-1 and -2 continuous predictor variables were mean-centered. In all the reported analyses, we included both actor and partner variables for all the predictors (all the relevant results are unchanged if only the partner variables are introduced as predictors). Because of the small sample size (i.e., 35 and 34 individuals, respectively, embedded in 18 couples for the life satisfaction and hedonic balance analyses, respectively), we report the non-robust standard error regression coefficient estimates, which are preferred in this case for interpretational purposes (all the reported effects remain significant if using the robust standard error estimates).

*Hedonic balance*. Similar to Study 1, we found no evidence that an actor's proficiency in identifying positive or negative emotions would be a significant predictor of either his/her own or his/her partner's hedonic balance (all *p*s > 0.15).

*Satisfaction with life*. Results of two separate sets of analyses revealed that among PD patients and their spouses, greater proficiency in identifying positive emotions did not exert a significant effect on spousal satisfaction with life, *b* = −0.30, *SE* = 0.51, *t*_(9)_ = −0.57, *p* = 0.58, even when accounting for years from PD onset, *b* = −0.23, *SE* = 0.14, *t*_(9)_ = −1.65, *p* = 0.13. Instead, with more years from PD symptom onset, it was proficiency in identifying negative emotions that became an increasingly stronger predictor of spousal satisfaction with life, *b* = 0.25, *SE* = 0.11, *t*_(9)_ = 2.22, *p* = 0.05 (for the full model, see Table 5). Follow-up analyses focused on discrete negative emotions revealed that it was greater expertise in identifying sadness that constituted an increasingly reliable predictor of greater spousal life satisfaction with more years from PD symptom onset, *b* = 0.22, *SE* = 0.06, *t*_(9)_ = 3.45, *p* < 0.01 (for the full model, see Table 6). Results of similar analyses involving expertise in identifying fear or anger failed to reach traditional levels of statistical significance (all *p*s > 0.21). Likewise, there was no evidence that an actor's proficiency in identifying either positive or negative emotions would be a significant predictor of his/her own life satisfaction levels (all *p*s > 0.24).

**Table 5:** **Parameter estimates for the HLM analyses predicting an actor's hedonic balance and life satisfaction from his/her partner's proficiency in identifying negative emotions in Study 2**.

**Fixed effect**	**Coefficient**	***SE***	***t*-value (dfs)**
**OUTCOME: ACTOR_SATISFACTION_WITH_LIFE**			
**For overall INTERCEPT,** β_0_			
Intercept2, B_00_	4.66	0.19	24.87(16)^**^
PD_Symptom_Onset, B_01_	0.09	0.05	1.73(16)
**For ACTOR_GENDER slope,** β_1_			
Intercept2, B_10_	−0.33	0.12	−2.78(9)^*^
PD_Symptom_Onset, B_11_	−0.05	0.03	−1.85(9)
**For ACTOR_PATIENT_STATUS slope,** β_2_			
Intercept2, B_20_	−0.48	0.13	−3.78(9)^**^
PD_Symptom_Onset, B_21_	0.09	0.03	3.09(9)^*^
**For PARTNER_NEGATIVE_EMOTIONS_RECOGNITION slope,** β_3_			
Intercept2, B_30_	0.34	0.39	0.89(9)
PD_Symptom_Onset, B_31_	0.25	0.11	2.22(9)*
**For ACTOR__NEGATIVE_EMOTIONS_RECOGNITION slope,** β**4**			
Intercept2, B_40_	0.48	0.39	1.23(9)
PD_Symptom_Onset, B_41_	−0.03	0.11	−0.31(9)

* p < 0.05;

** *p < 0.01*.

*The estimates are the non-robust error estimates (since they are preferred for interpretative purposes)*.

**Table 6:** **Parameter estimates for the HLM analyses predicting an actor's hedonic balance and life satisfaction from his/her partner's proficiency in identifying sadness in Study 2**.

**Fixed effect**	**Coefficient**	***SE***	***t*-value (dfs)**
**OUTCOME: ACTOR_SATISFACTION_WITH_LIFE**			
**For overall INTERCEPT,** β_0_			
Intercept2, B_00_	4.61	0.17	27.71(16)^**^
PD_Symptom_Onset, B_01_	0.08	0.05	1.68(16)
**For ACTOR_GENDER slope,** β_1_			
Intercept2, B_10_	−0.21	0.09	−2.38(9)^*^
PD_Symptom_Onset, B_11_	−0.07	0.02	−3.27(9)^**^
**For ACTOR_PATIENT_STATUS slope,** β_2_			
Intercept2, B_20_	−0.41	0.09	−4.37(9)^**^
PD_Symptom_Onset, B_21_	0.08	0.02	3.34(9)^**^
**For PARTNER_SADNESS_RECOGNITION slope,** β_3_			
Intercept2, B_30_	0.44	0.23	1.95(9)
PD_Symptom_Onset, B_31_	0.22	0.06	3.45(9)^**^
**For ACTOR_SADNESS_PERCEPTIONRECOGNITION slope,** β**4**			
Intercept2, B_40_	0.41	0.23	1.82(9)
PD_Symptom_Onset, B_41_	0.04	0.06	0.65(9)

* p < 0.05;

** *p < 0.01*.

*The estimates are the non-robust error estimates (since they are preferred for interpretative purposes)*.

*Patient-spouse differences*. Finally, we tested whether expertise in decoding positive vs. negative emotions would exert differential effects on spousal life satisfaction or hedonic balance, as a function of whether the spouse is the PD patient or not. To this end, we introduced the interaction between patient status and positive vs. negative emotion recognition scores as a level-1 predictor of partner well-being. Results of this set of analyses provided no support to the hypothesis that partner expertise in decoding positive vs. negative emotions would impact differentially either the life satisfaction or hedonic balance of the PD patients vs. that of their spouses (all *p*s > 0.37).

## General discussion

The present research investigated the link between expertise in reading postural cues of positive vs. negative emotions and spousal well-being among neurologically intact elderly couples (Study 1) and a sample of PD patients and their spouses (Study 2). Study 1 provided evidence that among healthy controls, greater expertise in identifying positive, rather than negative, emotions is linked to greater spousal cognitive, but not affective, well-being. Despite its specificity (see discussion below), such an association is broadly in line with our hypothesis that greater expertise in decoding positive emotional cues renders one better skilled at reading his/her spouse's emotional reactions during positive events and, thus, better able to provide adequate support to the spouse, which, in turn, is a well-documented contributor to spousal well-being (cf. Gable et al., 2012).

Study 2 extended the Study 1 findings regarding the unique link between spousal proficiency in emotion processing and an actor's cognitive, rather than affective, well-being levels. Specifically, in line with our hypotheses, Study 2 documented that under conditions that may render a close partner's negative emotions particularly informative for adjusting behavior to protect the dyadic environment, it is proficiency in reading negative, but not positive, emotions that predicts greater spousal life satisfaction. Thus, with more years from PD symptom onset, and, thus, conceivably, greater patient disability and care giver burden, it was proficiency in recognizing negative emotions, most importantly, sadness, that predicted greater spousal life satisfaction among PD patients and their partners. Moreover, although we did not detect any deficits in deciphering postural emotional cues among PD patients, we found suggestive evidence of PD-induced adaptation effects among the patients' spouses. Specifically, complementing earlier findings on the PD patients' deficits in producing facial expressions of positive emotions (Pitcairn et al., 1990; Simons et al., 2004), we found evidence of greater proficiency in decoding whole-body cues of happiness among PD spouses, relative to controls. Given the significance of positive emotion recognition for spousal well-being, documented in Study 1, it seems plausible that the PD spouses' advantage in identifying happiness would reflect a compensation mechanism, whose function would be to preserve the dyadic homeostasis. Further evidence suggestive of adaptation effects among PD spouses is provided by our findings that although in earlier stages, PD spouses may be somewhat less skilled than controls at negative emotion recognition, this deficit reverses with more years from PD symptom onset. Such an effect is indeed noteworthy, since among PD patients and their spouses, proficiency in identifying negative emotions becomes an increasingly reliable predictor of spousal satisfaction with life with more years from symptom onset.

Our present findings suggest several venues for future research on affective proficiency and well-being among married couples. First, the unique role of superior positive emotion recognition in spousal life satisfaction needs to be probed in future studies. For example, a spouse who is better skilled at decoding positive emotions may provide more effective support during propitious times and thus foster an actor's life satisfaction because s/he is better able to facilitate meaning-making processes that integrate an isolated positive event in the context of an actor's broader life goals and strivings. Complementarily, any beneficial effects of spousal positive emotion expertise on an actor's hedonic balance may be only indirect, mediated by changes in the actor's life satisfaction. To the best of our knowledge, there have been no investigations of the unique effects of effective social support on spousal affective vs. cognitive well-being. Consequently, future studies, assessing individual differences not only in well-being and positive emotion processing, but also in reappraisal and meaning making within a dyadic context, are needed to test the viability of our proposed hypotheses.

Second, the mechanisms underlying our observed association between performance on a point light walker task and spousal life satisfaction deserve further investigation. Prior research suggested that individual differences in decoding postural emotional cues are predictive of broader sociocognitive functioning (e.g., false belief reasoning, Phillips et al., 2011). Consequently, the question arises whether the observed link between spousal well-being and performance on the point light walker task is merely due to the fact that the latter is a good indicator of social cue understanding and utilization or whether indeed the ability to read whole-body (rather than facial or auditory, for example) emotional cues is particularly relevant to interpersonal functioning. With respect to the latter hypothesis, it seems indeed plausible that postural affective cues may be more informative to social exchanges than facial cues, because the latter may be easier to control and thus be used to conceal one's inner experience from others. Future studies using a comprehensive sociocognitive assessment package and individual differences or situational manipulations of emotional expression are needed to shed light on this issue.

Third, future studies should examine the implications of our findings that spouses are assorted not only on life satisfaction (cf. Bookwala and Schulz, 1996; Schimmack and Lucas, 2010), but also on traits predictive of life satisfaction, either on the interpersonal (i.e., happiness recognition) or intrapersonal (i.e., fear recognition) level (cf. Study 1). Indeed, in Study 1, we documented the association between expertise in identifying positive emotions and spousal life satisfaction, whereas others provided evidence that fear recognition plays an important role in determining prosocial behavior (for a review, see Niedenthal and Brauer, 2012). It thus seems plausible that the previously documented spousal matching on life satisfaction may be (partly) due to spousal assortment on dispositions that predict behaviors conducive to close others' happiness (i.e., proficiency in positive emotion recognition) or are linked to more fulfilling interpersonal exchanges (i.e., proficiency in fear recognition) and, thus, indirectly, contribute to one's own satisfaction with life. Future studies, examining the early acquaintanceship stages of potential romantic partners, may be required to test this hypothesis.

Fourth, additional research is needed to elucidate the underlying mechanisms and functionality of the PD spouses' proficiency in reading postural cues of positive emotions, as documented in Study 2 of the present manuscript. One venue for future research would be to shed light on whether PD spouses exhibit proficiency in decoding positive emotions across all modalities, or whether their advantage is more modality-specific (i.e., only detectable for non-facial forms of positive emotion expression). Such a distinction is important to be made, since it may also elucidate the pattern of expressive deficits associated for PD. Indeed, support for a more modality-specific (rather than modality-general) advantage in decoding positive affective cues among the PD spouses may suggest that, at least in the earlier stages of the disease, PD patients' ability to express emotions through non-facial cues may be relatively less affected. Nevertheless, future studies are needed to test these hypotheses by incorporating more modality-diverse emotional stimuli and by assessing PD patients' ability to produce and their spouses' proficiency in decoding positive emotional cues across multiple modalities. Likewise, future research should examine the mechanisms underlying the PD spouses' decreasing accuracy in decoding positive emotional cues and increasing accuracy in identifying negative emotional cues with more years from PD symptom onset. For example, one possibility may be that these effects are due to the decreased motivational salience of positive and increased relevance of negative emotional cues (cf. Study 2's link between spousal life satisfaction and proficiency in identifying negative emotions). Alternatively or additionally, in line with the documented PD-related impairments in producing positive emotional cues (Pitcairn et al., 1990; Simons et al., 2004), these effects may arise due to the PD spouses' diminishing exposure to positive emotional cues and relatively heightened exposure to negative emotional cues with more years from PD symptom onset.

Finally, the link between positive vs. negative emotion proficiency and spousal well-being deserves further investigation in more demographically diverse samples. Specifically, it is worth pointing out that our neurologically intact sample was exclusively comprised of older adults, who have been previously shown to exhibit greater sensitivity to positive, and lower sensitivity to negative, emotional stimuli, relative to younger adults (for a review, see Mather and Carstensen, 2005). Consequently, it may be the case that the null effect of proficiency in negative emotion recognition on spousal well-being may be partly due to older adults' superior ability to fend off negative emotions on their own, thereby minimizing the impact of any spousal aide, and/or their reduced tendency to allow negative emotions, evoked by external stressors, to permeate their marital interactions (for evidence on more positive marital interactions in older adulthood, see Levenson et al., 1994). In contrast, among younger adults, who presumably exhibit higher sensitivity to negative emotional stimuli (Mather and Carstensen, 2005), significantly greater interpersonal costs may be incurred by poorer negative emotion recognition and, thus, arguably, poorer ability to provide adequate support to a close partner during inauspicious times. Thus, although extant research suggests that social support provision to a close partner during propitious, rather than adverse, periods is a stronger contributor to the latter's well-being (Study 2, Gable et al., 2012), reduced ability to identify negative emotions and, consequently, respond appropriately to a partner during negative events may still result in significant hedonic damage, among younger, rather than elderly, social support recipients.

### Limitations

Inevitably, our present research has a few limitations. First, our correlational design precludes any conclusions regarding the causal direction of the link between proficiency in identifying positive vs. negative emotions and spousal life satisfaction. For example, in Study 1, it is plausible that individuals with higher, relative to lower, life satisfaction levels may express positive emotions more frequently. Thus, their spouses may have greater exposure to positive emotional cues and, consequently, gain greater expertise in decoding them. Nevertheless, if that were to be the case, then we would expect a stronger relationship between an actor's proficiency in decoding happiness and spousal hedonic balance, rather than life satisfaction. Importantly, this is not what we found in Study 1, where, in fact, the association between a partner's hedonic balance and an actor's ability to read happiness failed to reach conventional levels of statistical significance. Moreover, if frequency of exposure to a spouse's positive/negative emotional cues accounts for the link between an actor's proficiency in decoding emotional cues and spousal life satisfaction, then the Study 2 findings would suggest that among PD patients and their spouses, higher life satisfaction individuals express negative emotions more frequently, thereby fostering their spouses' expertise in deciphering them. Although we argued that expression of negative emotions is informative within a care giving context (cf. Monin and Schulz, 2009; Monin et al., 2009), we would find it difficult to contend that more frequent expression of such emotions is the hallmark of greater happiness among PD patients and their spouses. Nevertheless, future longitudinal studies, assessing the emotion expressive habits of both spouses, as well as their emotion expertise and well-being, are needed to elucidate the causal direction of the link between emotion recognition expertise and spousal well-being.

Second, we did not find any evidence of deficits in reading postural emotional cues among PD patients. To the best of our knowledge, there are no reports of PD-induced impairments in decoding whole-body affective cues in the literature, which renders our present null findings rather uncontroversial. Moreover, even deficits in facial emotion recognition have not been consistently documented among PD patients. Indeed, studies using subtler facial emotional cues and more advanced PD patients, tested on regular dosing schedules—as in our research—either failed to document any impairments (Adolphs et al., 1998; Sprengelmeyer et al., 2003) or documented impairments in disgust recognition (Suzuki et al., 2006; Assogna et al., 2010), an emotion not assessed in our study. Future studies with larger and more diverse PD samples, tested both on and off medication, may be required to elucidate the nature of any potential deficits in deciphering postural emotional cues among PD patients.

Third, although we posited that emotion recognition abilities are crucial to an individual's capacity to provide responsive support to a spouse, our research did not include any social support measures. Future studies, incorporating indicators of both enacted and perceived support during positive and negative events, are needed to characterize the hypothesized link between proficiency in identifying emotional cues and responsive support provision in close relationships.

Fourth, our research focused on a patient population exhibiting irreversibly increasing levels of disability. Future studies, examining care recipients with reversible or stable impairments, are warranted to shed light on any differential effects of spousal expertise in positive vs. negative emotion recognition as a function of disability type.

Fifth, in our present research, we used a rather narrowly defined standard of emotion recognition accuracy, specifically, the mean ratings provided by the neurologically intact sample in response to the point light walker clips. We regarded our operationalization of accuracy as justifiable because, beyond any idiosyncratic effects, a significant predictor of whether an actor would successfully decode his/her spouse's emotions is, arguably, his/her adherence to the affective vocabulary of those most demographically similar to his/her spouse. Nevertheless, future studies, using a broader affective vocabulary, based on the judgments of more demographically diverse samples, are certainly warranted to elucidate the nature of the association between an actor's emotion recognition proficiency and his/her spouse's well-being.

Finally, although the dynamic whole-body emotional stimuli, used in the present research, are more naturalistic than the static facial emotional stimuli, often employed in emotion recognition studies, they still fall short of the affective richness that typifies numerous interpersonal exchanges. For example, in each point light walker clip, the actor was requested to portray a specific emotion (see Heberlein and Saxe, 2005; Atkinson et al., 2007). Such affective “single-mindedness” is probably seldom characteristic of social actor's experiences in real life. Consequently, future studies are needed to elucidate the effect of proficiency in decoding emotions from realistic social interactions on spousal and dyadic well-being.

In conclusion, our present research suggests that expertise in decoding emotional cues may have wide-ranging implications for dyadic well-being. Moreover, our results imply that positive vs. negative emotions may play distinct roles in close relationship dynamics as a function of the spouses' neurological status and disability trajectory. Research is now needed to shed light on the mechanisms underlying the observed links between emotion recognition proficiency and close partner well-being across the lifespan of an intimate partnership.

### Conflict of interest statement

The authors declare that the research was conducted in the absence of any commercial or financial relationships that could be construed as a potential conflict of interest.
